# In vivo bioluminescence imaging for leptomeningeal dissemination of medulloblastoma in mouse models

**DOI:** 10.1186/s12885-016-2742-y

**Published:** 2016-09-08

**Authors:** Seung Ah Choi, Pil Ae Kwak, Seung-Ki Kim, Sung-Hye Park, Ji Yeoun Lee, Kyu-Chang Wang, Hyun Jeong Oh, Kyuwan Kim, Dong Soo Lee, Do Won Hwang, Ji Hoon Phi

**Affiliations:** 1 Division of Pediatric Neurosurgery, Seoul National University Children’s Hospital, 101 Daehakro, Jongno-gu, 110-744 Seoul Republic of Korea; 2 Department of Pathology, Seoul National University Hospital, College of Medicine, Seoul, Korea; 3 Department of Anatomy, Seoul National University College of Medicine, Seoul, Korea; 4 Department of Nuclear Medicine, Seoul National University College of Medicine, Seoul, Korea; 5 Department of Molecular Medicine and Biopharmaceutical Sciences, Graduate School of Convergence Science and Technology, and College of Medicine or College of Pharmacy, Seoul, Korea

**Keywords:** Medulloblastoma, Leptomeningeal seeding, Intracisternal injection, In vivo bioluminescence imaging

## Abstract

**Background:**

The primary cause of treatment failure in medulloblastomas (MB) is the development of leptomeningeal dissemination (seeding). For translational research on MB seeding, one of the major challenges is the development of reliable experimental models that simulate the seeding and growth characteristics of MBs. To overcome this obstacle, we improved an experimental mouse model by intracisternal inoculation of human MB cells and monitoring with in vivo live images.

**Methods:**

Human MB cells (UW426, D283 and MED8A) were transfected with a firefly luciferase gene and a Thy1.1 (CD90.1) marker linked with IRES under the control of the CMV promoter in a retroviral DNA backbone (effLuc). The MB-effLuc cells were injected into the cisterna magna using an intrathecal catheter, and bioluminescence images were captured. We performed histopathological analysis to confirm the extent of tumor seeding.

**Results:**

The luciferase activity of MB-effLuc cells displayed a gradually increasing pattern, which correlated with a quantitative luminometric assay. Live imaging showed that the MB-effLuc cells were diffusely distributed in the cervical spinal cord and the lumbosacral area. All mice injected with UW426-effLuc, D283-effLuc and MED8A-effLuc died within 51 days. The median survival was 22, 41 and 12 days after injection of 1.2 × 10^6^ UW426-effLuc, D283-effLuc and MED8A-effLuc cells, respectively. The histopathological studies revealed that the MB-effLuc cells spread extensively and diffusely along the leptomeninges of the brain and spinal cord, forming tumor cell-coated layers. The tumor cells in the subarachnoid space expressed a human nuclei marker and Ki-67. Compared with the intracerebellar injection method in which the subfrontal area and distal spinal cord were spared by tumor cell seeding in some mice, the intracisternal injection model more closely resembled the widespread leptomeningeal seeding observed in MB patients.

**Conclusion:**

The results and described method are valuable resources for further translational research to overcome MB seeding.

## Background

Medulloblastoma (MB) is a malignant childhood brain tumor that develops in the cerebellum and brainstem. MB commonly spreads through the cerebrospinal fluid (CSF) and disseminates to the surfaces of the brain and spinal cord. The overall 5-year survival rate of patients with MB is approximately 70 % [1, 2]. Many studies have demonstrated that tumor dissemination (seeding) into the cerebrospinal fluid may have the strongest impact on patient prognosis [3, 4].

Recently, genomic studies have revealed novel characteristics of MBs, including molecular subtypes and driver mutations. However, relatively little has is known about the mechanisms of MB seeding. The paucity of metastatic MB tissues obtained by surgical biopsy may be the chief problem in studying MB seeding. Therefore, establishing and refining a stable MB seeding animal model would be of great use to enhance translational research for this clinically challenging issue.

There are many types of transgenic mouse MB models with a high rate of spontaneous tumor development. However, the majority of transgenic MB mouse models develop non-disseminated tumors [5]. An MB mouse model with frequent tumor seeding has been introduced; however, the mouse model relied on random transposon mutagenesis in multiple transgenic backgrounds, and tumor seeding was observed in only 40 % of mice [6].

Tumor xenograft models using human MB cell lines have several advantages, including technical accessibility, short tumor growth time, and reliable development of tumor seeding. Several methods have been described to establish the MB seeding model and involve transplanting human MB cells into the mouse cerebrum [7], cerebellum [8], subdural space [9], or cisterna magna [4, 10, 11]. However, each method possesses some limitations, such as the risks of surgical techniques, complexity of quantitative analysis in live animals, and difficulties of precisely simulating MB characteristics. Therefore, a more stable and accurate method is required to establish a reliable MB seeding model. For a long-term follow-up in vivo study, bioluminescence imaging (BLI) provides accurate and reliable results about tumor growth patterns [12, 13]. BLI also allows longitudinal follow-up for further therapeutic intervention by enabling repetitive examinations [12, 13].

In this study, we demonstrated a modified xenograft MB mouse model by injecting tumor cells into the cisterna magna. We monitored the MB seeding state by in vivo live imaging using a bioluminescent signal for accurate quantitative analysis. We compared the efficiency of several widely used MB cell lines to establish MB seeding models. The efficiency of this method to make a MB seeding model was compared to the result using the intracerebellar injection method. The method and results of this study are useful resources for further translational research on MB dissemination.

## Methods

### Cell cultures

Two human MB cell lines (UW426 and MED8A) were generously provided by Dr. Young Shin Ra (Asan Medical Center, Seoul, Korea). D283 cells were purchased from the American Type Culture Collection (ATCC, Manassas, VA). UW426 and MED8A were maintained in Dulbecco’s Modified Eagle’s Medium (DMEM; Invitrogen, Carlsbad, CA) with 10 % fetal bovine serum (FBS; Invitrogen) and 1× penicillin-streptomycin (P/S; Invitrogen). D283 cells were maintained in Eagle’s Minimum Essential Medium (EMEM; ATCC) with 10 % FBS and 1× P/S (Invitrogen). All cells were incubated at 37 °C in a 5 % CO_2_/ 95 % air atmosphere.

### Retroviral infection

MB cells (UW426, D283 and MED8A) overexpressing the enhanced firefly luciferase gene (effLuc) were generated by retroviral infection. The DNA backbone of the retroviral vector contained a Thy1.1 (CD90.1) marker and the effLuc gene linked to an internal ribosome entry site (IRES) under the control of the cytomegalovirus (CMV) promoter. HEK293FT cells were co-transfected with an effLuc viral vector and a DNA vector carrying major structural proteins (GAG, Pol, and Env) using Lipofectamine 2000 (Invitrogen). Supernatants were collected after 48 h, filtered (0.2 μm), and added to target MB cells with 10 mM polybrene. The infected cells were sorted by magnetic-activated cell sorting (MACS; Miltenyi Biotech Ltd., Bergisch Gladbach, Germany) using a monoclonal anti-CD90.1 conjugated to magnetic microbeads.

### In vitro luciferase assay

MB-effLuc (UW426-effLuc, D283-effLuc and MED8A-effLuc) cells were seeded into a 6-well plate, washed with PBS, and lysed with lysis buffer (Promega, Madison, WI). The cell lysates were collected and redistributed into a 96-well plate. The luciferase assay was performed using a luciferase assay kit (Promega). The bioluminescence intensity of each cell lysate was measured using a microplate luminometer (GLOMAX; Promega) at an acquisition time of 1 s. Each luciferase assay was performed three times with three replicates per group.

### Mouse model of MB seeding

Female BALB/c-nude mice (7–8 weeks; Orient Bio Inc., Seongnam, Korea) were housed under specific pathogen-free conditions. All animal experiments were performed at Seoul National University Hospital (SNUH) Biomedical Research Institute (Seoul, Korea) with the approval of the Institutional Animal Care and Use Committee (SNUH-IACUC No.14-0206-S1A2). Prior to cell injection, the mice were anesthetized with an intraperitoneal injection of 20 mg/kg Zoletil and 10 mg/kg xylazine. We used two different injection methods for the MB seeding model.

First, we slowly injected the MB-effLuc cells into the cisterna magna. MB-effLuc cells were suspended in 25 μl of PBS at different concentrations (UW426, 1.2 × 10^6^ cells; D283, 1.2 × 10^6^ cells; MED8A, 1.2 × 10^5^ to 1.2 × 10^6^ cells). The head of the mouse was bent (at an angle of approximately 90° to the body) in a stereotaxic frame (Harvard Apparatus, Holliston, MA), the skin over the posterior atlanto-occipital membrane was cut, and the muscular layers were moved aside as previously described [14]. The tip of a Hamilton syringe (30 gauge; Hamilton, Reno, NV) was connected to an intrathecal catheter (ALZET Osmotic Pumps, Cupertino, CA) and filled with the MB-effLuc cells. The tip of the intrathecal catheter was carefully inserted into the cisterna magna through the posterior atlanto-occipital membrane, and the cells were injected at a rate of 1 μl/min using an infusion pump (Harvard Apparatus, Holliston, MD). The muscular layers were realigned, and the skin was sutured.

Second, we slowly injected the UW426-effLuc (1.2 × 10^5^) cells through the burr hole into the right cerebellar hemisphere (1 mm to the right of the midline, 1 mm posterior to the coronal suture, and 3 mm deep) as previously described [8]. The mice were monitored daily for neurologic symptoms until they were euthanized, at which time their brains and spinal cords were removed for histopathological analysis.

### In vivo bioluminescence imaging (BLI)

For BLI acquisition, D-Luciferin (150 mg/kg, Caliper Life Sciences, Hopkinton, MA, USA) was prepared and injected intraperitoneally following the manufacturer’s protocol. After anesthesia with 2 % isoflurane in 100 % O_2_ through a nose cone, images were captured using an IVIS-100 imaging system (Xenogen Corp., Alameda, CA) every 3 days post-transplantation. Images were acquired by integrating light for 1–3 min, and the luminescence intensity in regions of interest from each image was quantified to examine the viability of the implanted cells.

### Histological and immunofluorescence analysis

At the time of sacrifice, the mice were perfused with saline containing 2.5 U/ml heparin and fixed with 4 % paraformaldehyde under deep anesthesia. The brains and spinal cords were decalcified with 10 % EDTA (pH 7.2) for 2 weeks and dehydrated in a graded sucrose series. The tissues were embedded in OCT compound (Tissue-Tek®; Sakura, Tokyo, Japan) and stored at −80 °C. Ten micron-thick frozen blocks were sagittally sectioned using a cryostat. The sections were stained with hematoxylin and eosin.

Sectioned tissues were immunostained with anti-human nuclei (1:250; Millipore, Billerica, MA) and/or anti-Ki67 (1:400; Abcam, Cambridge, UK) antibodies as previously described [4].

### Statistical analysis

Statistical significance was calculated using SPSS version 21.0 software (IBM, Armonk, NY). The survival data were presented as Kaplan-Meier plots and analyzed by the log-rank test. *P*-values < 0.05 were considered statistically significant.

## Results

### Establishment of medulloblastoma (MB)-effLuc cells

**Fig. 1:**
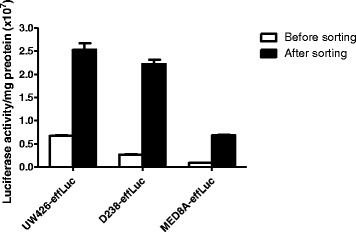
Retroviral construct that contains the effLuc gene and Thy1.1 (CD90.1), linked with an internal ribosomal entry site (IRES). The luciferase activity of effLuc cells cultured in a 96-well plate was measured using an IVIS-100 optical imaging device. Firefly luciferase activity continuously increased in effLuc cells in proportion to cell number

To visualize in vivo characteristics of MB cells, three different MB cell lines were infected with a retroviral vector containing the enhanced firefly luciferase (effLuc) reporter gene. The cells that possess effLuc genes among a heterogeneous cell population were selectively sorted by a magnetic-activated cell separator using anti-CD90.1-conjugated magnetic microbeads. All MB-effLuc (UW426-effLuc, D283-effLuc and MED8A-effLuc) cells showed significantly enhanced luciferase signals (approximately 4, 9, 8-fold increase, respectively) after magnetic cell sorting (Fig. 1).

### MB-effLuc seeding model and survival analysis

**Fig. 2:**
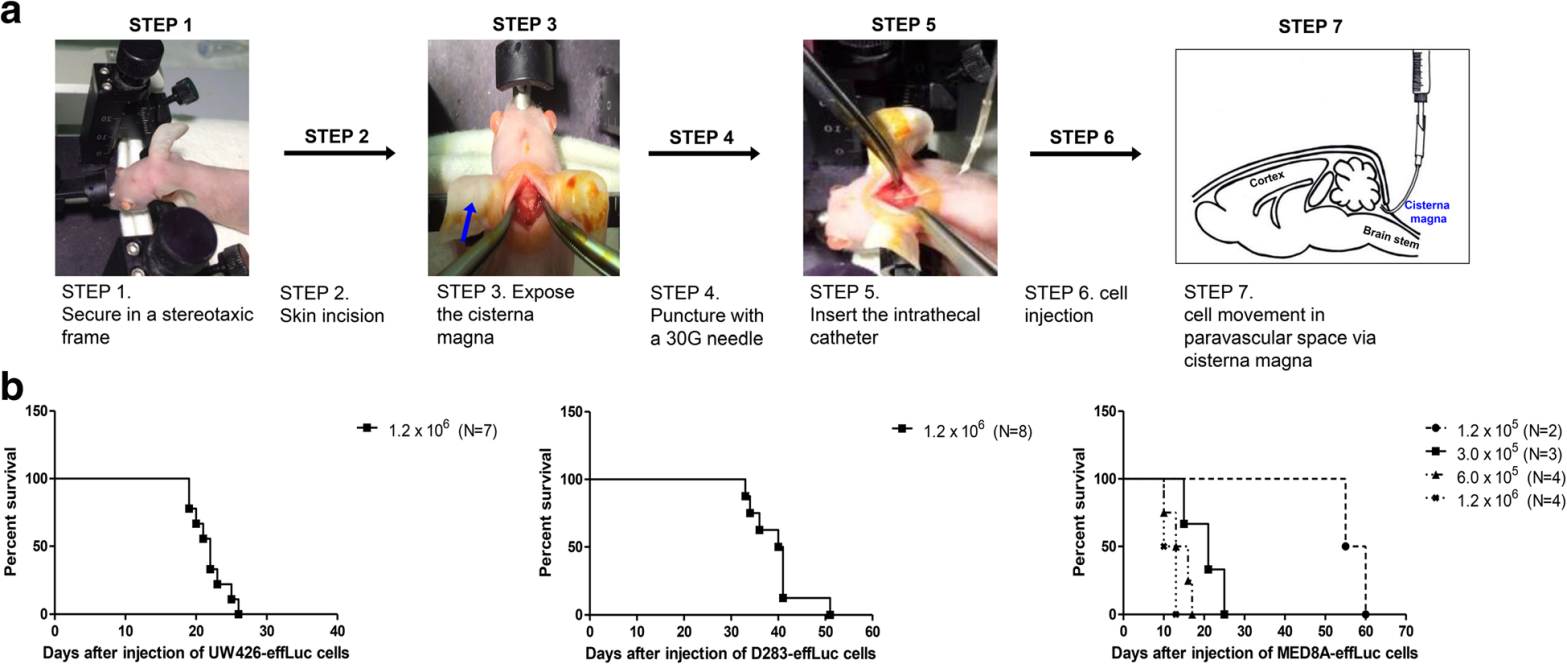
Intracisternal injection for medulloblastoma (MB) seeding model and survival analysis. **a** Immune-deficient BALB/c-nude mice were mounted on the stereotactic device. The mouse heads were fixed in a stereotactic device, and the cisterna magna was exposed. MB-effLuc cells (UW426-effLuc: 1.2 × 10^6^; MED8A-effLuc: 1.2 × 10^5^, 3.0 × 10^5^, 6.0 × 10^5^ and 1.2 × 10^6^; D283-effLuc: 1.2 × 10^6^) were slowly injected into the subarachnoid space of the cisterna magna. **b** The median survival days of each group were estimated: UW426-effLuc: 22 days for 1.2 × 10^6^ cells; D283-effLuc: 41 days for 1.2 × 10^6^ cells; MED8A-effLuc: 57 days for 1.2 × 10^5^ cells, 21 days for 3.0 × 10^5^ cells, 14 days for 6.0 × 10^5^ cells and 12 days for 1.2 × 10^6^ cells

The mouse seeding model was created by injecting the three different MB-effLuc (UW426-effLuc, 1.2 × 10^6^, *N* = 7; D283-effLuc: 1.2 × 10^6^, *N* = 8; MED8A-effLuc, 1.2 × 10^5^, *N* = 2; 3.0 × 10^5^, *N* = 3; 6.0 × 10^5^, *N* = 4; and 1.2 × 10^6^, *N* = 4) cells into the subarachnoid space of the cisterna magna as shown in Fig. 2a. The survival rates of each group were calculated. The median survival was 22 and 41 days for the UW426-effLuc (1.2 × 10^6^) and D283-effLuc (1.2 × 10^6^) cells, respectively (Fig. 2b). After MED8A-effLuc (1.2 × 10^6^) cell injection, the mice began to die at 10 days (median survival of 12 days). We changed the number of injected MED8A-effLuc cells to determine the optimal loading numbers, and the median survival was 14 days for 6.0 × 10^5^ cells, 21 days for 3.0 × 10^5^ cells, and 57 days for 1.2 × 10^5^ cells (Fig. 2b). Therefore, we concluded that 3.0 × 10^5^ MED8A-effLuc cells were the optimal cell number for further studies with some therapeutic intent.

### In vivo bioluminescence imaging (BLI) of the MB-effLuc seeding model

**Fig. 3:**
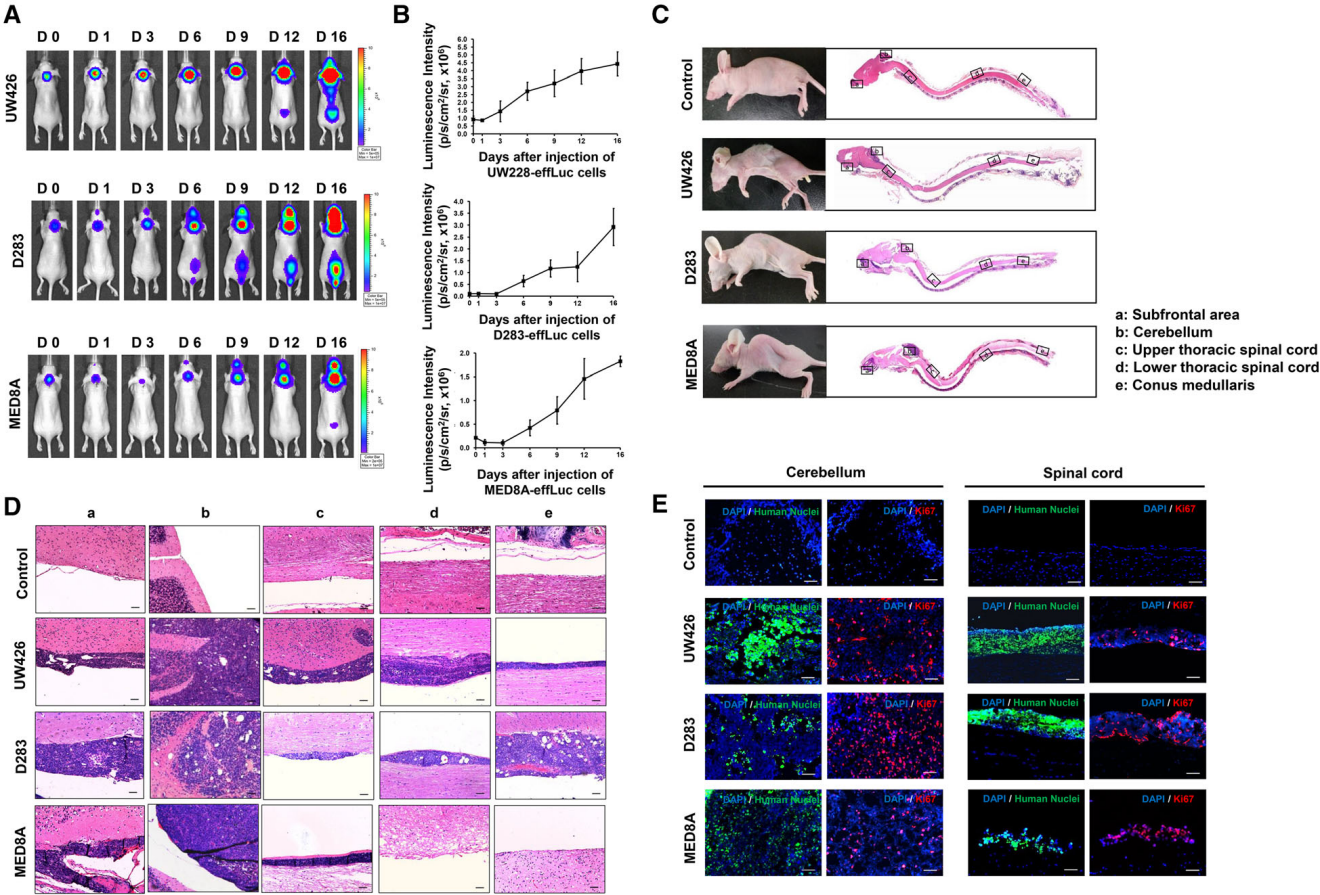
Bioluminescence imaging (BLI) and histological analysis for the evaluation of medulloblastoma (MB) seeding. (**A** and **B**) After injection of the MB-effLuc cells (UW426, 1.2 × 10^6^; D283, 1.2 × 10^6^; MED8A, 3.0 × 10^5^), firefly luciferase BLI was evaluated and quantified at the indicated days. (**C**) Representative longitudinal sections of the brains and spinal cords of the mice with saline (control) or MB-effLuc cells (hematoxylin & eosin stain) (1.25×). (**D**) Inlet figures denote the subfrontal area (a), cerebellum (b), upper thoracic spinal cord (c), lower thoracic spinal cord (d), and conus medullaris (e). Scale bars represent 50 μm. (**E**) Representative immunofluorescence images (DAPI: blue, Human Nuclei: green, Ki67: red) show that the MB-effLuc cells are highly proliferative in vivo. Scale bars represent 50 μm

After intracisternal injection of MB-effLuc cells (UW426-effLuc, 1.2 × 10^6^; MED8A-effLuc, 3.0 × 10^5^; D283-effLuc, 1.2 × 10^6^ cells, *N* = 5 for each group), BLI was acquired every 3–5 days. We observed bioluminescence signals at day 0 in all mice (Fig. 3a). The signals spread through the spinal axis and distributed to the lumbosacral area between day 1 and day 6 (Fig. 3a). Photon counts in the region of interest (ROI) were measured to quantify the BLI results. The BLI signal in mice that were injected with MB-effLuc cells increased gradually and diffused throughout the spinal canal. Cell proliferation in MB-effLuc cell groups was markedly enhanced after day 3 until day 16, and each cell growth pattern was clearly discriminated with different luminescence between day 3 [UW426: (1.43 ± 0.66) × 10^6^ p/s/cm^2^/sr; D283: (0.10 ± 0.05) × 10^6^ p/s/cm^2^/sr; MED8A: (0.10 ± 0.06) × 10^6^ p/s/cm^2^/sr] and day 16 [UW426: (4.43 ± 0.76) × 10^6^ p/s/cm^2^/sr; D283: (2.92 ± 0.78) × 10^6^ p/s/cm^2^/sr; MED8A: (1.82 ± 0.11) × 10^6^ p/s/cm^2^/sr; Fig. 3b].

### Histopathology of the MB seeding model

To confirm the presence of leptomeningeal tumor seeding, mice injected with UW426-effLuc, D283-effLuc, and MED8A-effLuc cells (*N* = 5 for each group) were sacrificed at 22, 41 and 12 days, respectively. Histological examination showed that tumor cells disseminated from the subfrontal area to the conus medullaris in mice injected with UW426-effLuc and D283-effLuc cells (Fig. 3c and d). Contrary to this widespread seeding pattern, large tumor masses were observed at the MED8A-effLuc cell injection site (Fig. 3c and d). No tumor seeding was found in lumbosacral areas in mice injected with MED8A-effLuc cells. Table 1 summarizes the dissemination pattern of each tumor cell group.

**Table 1:** Dissemination pattern of tumor seeding in each cell line group

Cell type	UW426-effLuc	D283-effLuc	MED8A-effLuc
Subfrontal area	5/5 (100 %)	5/5 (100 %)	1/3 (33 %)
Cerebellum	5/5 (100 %)	5/5 (100 %)	3/3 (100 %)
Upper thoracic spinal cord	5/5 (100 %)	5/5 (100 %)	3/3 (100 %)
Lower thoracic spinal cord	5/5 (100 %)	5/5 (100 %)	1/3 (33 %)
Conus medullaris	4/5 (80 %)	5/5 (100 %)	0/3 (0 %)

Tumor histology was reviewed by a neuropathologist (Park SH). Tumors formed by MB-effLuc cell injection consisted of a monomorphic population of small blue cells, with a high nucleo-cytoplasmic ratio, inconspicuous nucleoli, occasional cell wrapping, and frequent mitoses. With diffuse sheets of small, round, blue tumor cells, the tumors corresponded to the classic subtype of human MB. These cells developed small nucleoli (micronucleoli), scanty cytoplasm, and only subtle signs of differentiation. In accordance with the monomorphic nature of the cells, they maintained uniform morphology and neuropil-type stroma after extensive in vivo growth and spread along the leptomeninges (Fig. 3c and d). Immunofluorescence images revealed that the injected cells were widespread along the pial surface of the spinal cord, and they strongly expressed Ki-67 (Fig. 3e).

### Comparison of intracisternal injection with the intracerebellar injection method

**Fig. 4:**
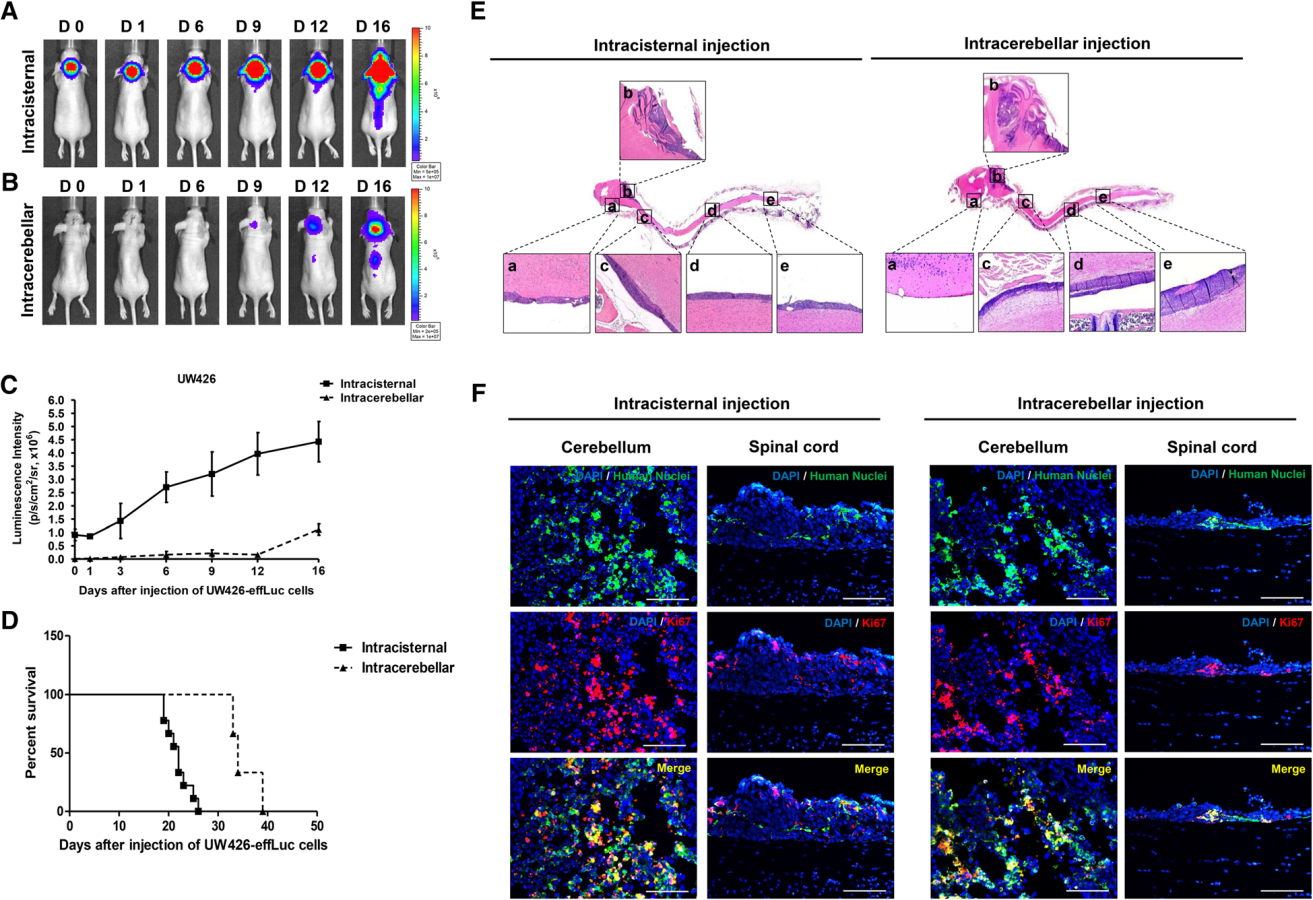
The intracisternal injection and intracerebellar injection methods. (**A**) BLI of mice with cell injection into the cisterna magna show that the signals are observed at day 0, expand at day 6, and begin to spread to the spinal cord at day 9. (**B**) The signals were detected first at day 6 and gradually migrated to the spinal cord from day 12 in mice with cells injected into the cerebellum. (**C**) BLI quantification of tumor-occupied areas during the study. (**D**) The median survival was 22 and 34 days in the intracisternal injection model and intracerebellar injection model, respectively. (**E**) Histopathology of xenograft MB seeding. Inlet figures denote the subfrontal area (a), cerebellum (b), upper thoracic spinal cord (c), lower thoracic spinal cord (d), and conus medullaris (e). The intracisternal injection model displays strong similarity to the histopathological character and widespread dissemination pattern of MB seeding. (**F**) Representative immunofluorescence images (DAPI: blue, Human Nuclei: green, Ki-67: red, Merge: yellow) show that both the intracisternal- and intracerebellar-injected UW426-effLuc cells are highly proliferative in vivo. Scale bars represent 50 μm

Intracisternal (1.2 × 10^6^ cells, *N* = 7) and intracerebellar injections (1.2 × 10^5^ cells, *N* = 5) using UW426-effLuc cells were performed. In mice that had injections into the cisterna magna, BLI signals were observed at day 0 and gradually spread along the spinal axis over time [day 0: (0.91 ± 0.49) × 10^6^ p/s/cm^2^/sr; day 16, (4.43 ± 0.76) × 10^6^ p/s/cm^2^/sr; Fig. 4a and c]. However, in mice that had injections into the cerebellar hemisphere, the signals were detected on day 9 [(0.16 ± 0.19) × 10^6^ p/s/cm^2^/sr], suddenly increased and migrated to the spinal cord from day 16 [(1.11 ± 0.21) × 10^6^ p/s/cm^2^/sr; Fig. 4b and c]. The median survival was 22 days for the intracisternal injection model and 34 days for the intracerebellar injection model (Fig. 4d). To analyze the histopathology, mice that had injections into the cisterna magna (*N* = 5) were sacrificed at 22 days and mice that had injections into the cerebellar hemisphere (*N* = 5) were sacrificed at 35 days. In the intracisternal injection model, tumor cells spread in the entire neuraxis from the subfrontal area and cerebellar surface to the distal spinal cord. In the intracerebellar injection model, tumor cells aggregated in the cerebellum and did not disseminate into the subfrontal area or distal spinal cord in some animals examined (Fig. 4e). Table 2 summarizes the dissemination pattern of each cell injection method. Immunofluorescence staining revealed that abundant human nuclei and Ki67 double positive cells were observed in mice that had injections into the cisterna magna or cerebellar hemisphere (Fig. 4f).

**Table 2:** Dissemination pattern of tumor seeding location by injection methods

Cell injection site	Intracisternal injection	Intracerebellar injection
Subfrontal area	5/5 (100 %)	1/5 (20 %)
Cerebellum	5/5 (100 %)	4/5 (80 %)
Upper thoracic spinal cord	5/5 (100 %)	5/5 (100 %)
Lower thoracic spinal cord	5/5 (100 %)	3/5 (60 %)
Conus medullaris	4/5 (80 %)	3/5 (60 %)

## Discussion

Because tumor seeding is the most troublesome problem in the treatment of human MBs, we have an urgent need to develop novel therapeutics to prevent or eliminate tumor seeding in these patients. Therefore, the establishment of an effective MB seeding model and the evaluation of the extent of tumor spread in live conditions are essential for studies on innovative therapeutics. For MB xenograft mouse models, four different injection routes have been introduced: intracerebral, subdural, intracerebellar, and intracisternal [4, 7, 9, 10]. Intracerebral and subdural (into the supratentorial subdural space) injections of MB cells have inherent limitations to identify MB characteristics in vivo because MB develops in or around the cerebellum and spreads via subarachnoid spaces. Intracerebellar MB xenograft models can be a good alternative. This model was used in many studies for the preclinical evaluation of medications for MB tumor cells in vivo [8]. One problem of intracerebellar injection can be an overgrowth of tumor masses and early death of mice before adequate tumor seeding develops. Intracisternal cell injection can circumvent this problem because the injected tumor cells readily disseminate through the CSF into the distant subarachnoid space. In the present study, a comparison of the intracerebellar and intracisternal injection methods using the same cell line revealed that tumor seeding was incomplete in some mice with intracerebellar cell injection despite the longer incubation period for the intracerebellar injection method.

In our previous study, we injected D283 MB cells labeled with fluorescent magnetic nanoparticles into the cisterna magna and monitored tumor seeding with in vivo live imaging [4]. Injection into the cisterna magna using a 30-gauge needle is a fastidious surgical technique that allows control over the injection speed. Although the fluorescent signals were quantifiable by measuring the area of signal intensity, the result was less sensitive in long-term observations because the signals decreased as tumor cells proliferated [12, 15–17]. To overcome these problems of previous seeding models, we established MB-effLuc cells and injected the cells into the cisterna magna using an intrathecal catheter. Intrathecal catheterization via the cistern magna was used for injection to the spinal fluid [18]. Bioluminescence images (BLI) were obtained every 3 days, and live mice were monitored until a humane endpoint when serious clinical symptoms of tumor seeding had developed. The signals increased and spread from the injection site to the distal spinal cord, confirming the establishment of stable MB seeding models.

We also compared the seeding pattern and median survival rates in 3 commonly used MB cell lines. The median survival was 22 and 41 days in UW426-effLuc (1.2 × 10^6^) and D283-effLuc (1.2 × 10^6^) cells, respectively. For MED8A-effLuc cells, the median survival was too short to apply further therapeutics with the same number of cells (1.2 × 10^6^). Therefore, a reduction in injection number to 3.0 × 10^5^ (median survival of 21 days) was necessary. In brain tumor xenograft models, setting the proper survival time is important for testing novel therapeutics. Shortening the median survival time may not provide sufficient intervention time, whereas overextension may be laborious and time-consuming. It is also not recommended to increase the number of cells in the seeding model to save tumor growth time. This method may result in the early death of the mice due to the compressive effect of tumor masses before the cells spread sufficiently through the CSF. Especially for the MB seeding model, the appropriate number of cells and injection site are important for widespread tumor seeding along the neuraxis while allowing the mice to survive.

## Conclusions

In this study, we confirmed that intracisternal injection of MB cells exhibited stronger similarity to the dissemination pattern of MB. Furthermore, we proposed several modifications to improve the technique of the MB seeding model and provided high-throughput in vivo imaging. The method and cell injection numbers described here are useful resources for future translational research of MB seeding.

## Abbreviations

ATCC: American type culture collection
BLI: Bioluminescence imaging
CSF: Cerebrospinal fluid
DMEM: Dulbecco’s modified eagle’s medium
effLuc: Firefly luciferase gene and a Thy1.1 (CD90.1) marker linked with IRES under the control of the CMV promoter in a retroviral DNA backbone
EMEM: Eagle’s minimum essential medium
FBS: Fetal bovine serum
IACUC: Institutional animal care and use committee
MACS: Magnetic-activated cell sorting
MB: Medulloblastomas
P/S: Penicillin-streptomycin
Seeding: Leptomeningeal dissemination
SNUH: Seoul National University Hospital

## References

[1] Phi JH, Lee J, Wang KC, Cho BK, Kim IO, Park CK (2011). Cerebrospinal fluid M staging for medulloblastoma: reappraisal of Chang’s M staging based on the CSF flow. Neuro-Oncology.

[2] Young RJ, Khakoo Y, Yhu S, Wolden S, De Braganca KC, Gilheeney SW (2015). Extraneural metastases of medulloblastoma: desmoplastic variants may have prolonged survival. Pediatr Blood Cancer.

[3] Jenkin D, Shabanah MA, Shail EA, Gray A, Hassounah M, Khafaga Y (2000). Prognostic factors for medulloblastoma. Int J Radiat Oncol Biol Phys.

[4] Phi JH, Choi SA, Lim SH, Lee J, Wang KC, Park SH (2013). ID3 contributes to cerebrospinal fluid seeding and poor prognosis in medulloblastoma. BMC Cancer.

[5] Goodrich LV, Milenkovic L, Higgins KM, Scott MP (1997). Altered neural cell fates and medulloblastoma in mouse patched mutants. Science.

[6] Wu X, Northcott PA, Dubuc A, Dupuy AJ, Shih DJ, Witt H (2012). Clonal selection drives genetic divergence of metastatic medulloblastoma. Nature.

[7] Kim SK, Kim SU, Park IH, Bang JH, Aboody KS, Wang KC (2006). Human neural stem cells target experimental intracranial medulloblastoma and deliver a therapeutic gene leading to tumor regression. Clin Cancer Res.

[8] Shu Q, Antalffy B, Su JM, Adesina A, Ou CN, Pietsch T (2006). Valproic Acid prolongs survival time of severe combined immunodeficient mice bearing intracerebellar orthotopic medulloblastoma xenografts. Clin Cancer Res.

[9] Lim SH, Choi SA, Lee JY, Wang KC, Phi JH, Lee DH (2011). Therapeutic targeting of subdural medulloblastomas using human neural stem cells expressing carboxylesterase. Cancer Gene Ther.

[10] Shimato S, Natsume A, Takeuchi H, Wakabayashi T, Fujii M, Ito M (2007). Human neural stem cells target and deliver therapeutic gene to experimental leptomeningeal medulloblastoma. Gene Ther.

[11] Chen Y, Imai H, Ito A, Saito N (2013). Novel modified method for injection into the cerebrospinal fluid via the cerebellomedullary cistern in mice. Acta Neurobiol Exp.

[12] Frampas E, Maurel C, Thedrez P, Remaud-Le Saec P, Faivre-Chauvet A, Barbet J (2011). The intraportal injection model for liver metastasis: advantages of associated bioluminescence to assess tumor growth and influences on tumor uptake of radiolabeled anti-carcinoembryonic antigen antibody. Nucl Med Commun.

[13] Smakman N, Martens A, Kranenburg O, Borel Rinkes IH (2004). Validation of bioluminescence imaging of colorectal liver metastases in the mouse. J Surg Res.

[14] Huda F, Konno A, Matsuzaki Y, Goenawan H, Miyake K, Shimada T (2014). Distinct transduction profiles in the CNS via three injection routes of AAV9 and the application to generation of a neurodegenerative mouse model. Mol Ther Methods Clin Dev.

[15] Dacres H, Michie M, Anderson A, Trowell SC (2013). Advantages of substituting bioluminescence for fluorescence in a resonance energy transfer-based periplasmic binding protein biosensor. Biosens Bioelectron.

[16] do Hwang W, Jin Y, do Lee H, Kim HY, Cho HN, Chung HJ (2014). In vivo bioluminescence imaging for prolonged survival of transplanted human neural stem cells using 3D biocompatible scaffold in corticectomized rat model. PLoS One.

[17] Rousseau J, Escriou V, Perrot P, Picarda G, Charrier C, Scherman D (2010). Advantages of bioluminescence imaging to follow siRNA or chemotherapeutic treatments in osteosarcoma preclinical models. Cancer Gene Ther.

[18] Rangroo Thrane V, Thrane AS, Plog BA, Thiyagarajan M, Iliff JJ, Deane R (2013). Paravascular microcirculation facilitates rapid lipid transport and astrocyte signaling in the brain. Sci Rep.

